# The Role of Genetics and Oxidative Stress in the Etiology of Male Infertility—A Unifying Hypothesis?

**DOI:** 10.3389/fendo.2020.581838

**Published:** 2020-09-30

**Authors:** Robert John Aitken, Mark A. Baker

**Affiliations:** ^1^Faculty of Science and Faculty of Health and Medicine, Priority Research Centre in Reproductive Science, University of Newcastle, Callaghan, NSW, Australia; ^2^Hunter Medical Research Institute, New Lambton Heights, NSW, Australia

**Keywords:** male infertility, mutation, oxidative stress, spermatozoa, DNA damage

## Abstract

Despite the high prevalence of male infertility, very little is known about its etiology. In recent years however, advances in gene sequencing technology have enabled us to identify a large number of rare single point mutations responsible for impeding all aspects of male reproduction from its embryonic origins, through the endocrine regulation of spermatogenesis to germ cell differentiation and sperm function. Such monogenic mutations aside, the most common genetic causes of male infertility are aneuploidies such as Klinefelter syndrome and Y-chromosome mutations which together account for around 20–25% of all cases of non-obstructive azoospermia. Oxidative stress has also emerged as a major cause of male fertility with at least 40% of patients exhibiting some evidence of redox attack, resulting in high levels of lipid peroxidation and oxidative DNA damage in the form of 8-hydroxy-2'-deoxyguanosine (8OHdG). The latter is highly mutagenic and may contribute to *de novo* mutations in our species, 75% of which are known to occur in the male germ line. An examination of 8OHdG lesions in the human sperm genome has revealed ~9,000 genomic regions vulnerable to oxidative attack in spermatozoa. While these oxidized bases are generally spread widely across the genome, a particular region on chromosome 15 appears to be a hot spot for oxidative attack. This locus maps to a genetic location which has linkages to male infertility, cancer, imprinting disorders and a variety of behavioral conditions (autism, bipolar disease, spontaneous schizophrenia) which have been linked to the age of the father at the moment of conception. We present a hypothesis whereby a number of environmental, lifestyle and clinical factors conspire to induce oxidative DNA damage in the male germ line which then triggers the formation *de novo* mutations which can have a major impact on the health of the offspring including their subsequent fertility.

## Introduction

Spermatogenesis is an immensely complicated process involving the coordinated action of thousands of genes in order to generate one of the most complex, specialized cell types in human biology, the spermatozoon. Given this complexity, it may not be surprising that “the male factor” is held to be a major contributor to human infertility, although, the extent of this contribution is still a matter for conjecture. A recent survey of papers that have set out to determine the causes of human infertility by assigning each case to one of four categories (male factor, female factor, both male and female factors, and unexplained) indicated that defects in the male were, on average (±SEM), thought to account for 21.1 ± 2.8% of all infertility, while the remaining causes distributed as follows: female factors (42.8 ± 3.2%), combined male and female factors (24.2 ± 4.9%) and unexplained infertility (13.2 ± 2.1%) (1). Examination of individual studies reveals a wide range of estimates for the incidence of male infertility (5–35%) that may reflect real differences between populations in terms of the quality of primary health care, occupational and environmental exposures to reproductive toxicants, age, dietary factors, obesity, climate, education, recreational exposure to drugs and genetic as well as epigenetic factors (1).

The major problem with all of these assessments is that the existence of male factor infertility was determined on the basis of a conventional semen profile. While the latter is acknowledged as a fundamental component of diagnostic andrology, with few exceptions, it is also widely understood that the criteria used to create such semen profiles (sperm count, motility and morphology) are not precisely predictive of infertility. In a prospective study of patients exhibiting unexplained infertility (normal female partner and normal conventional semen profile), these criteria were found be incapable of predicting the chances of spontaneous conception during a follow-up period lasting up to 4 years during which the patients received no further treatment (2). Similarly, in assisted conception cycles, including both *in vitro* fertilization (IVF) and intracytoplasmic sperm injection (ICSI) the conventional semen profile has been found to be of no value in predicting fertilization rates (3). However, when elements of the semen profile are combined with other data describing, for example, competence for sperm-oocyte fusion, the detailed movement characteristics of the spermatozoa and aspects of oxidative stress, then algorithms can be generated that better predict the relative fertility of males *in vitro* and *in vivo* (2, 4, 5). The exception to this rule is when the semen profile is seriously flawed, as is the case with azoospermia or severe oligozoospermia (<5 million/ml) where fertility rates are significantly reduced as might be anticipated (6). At the same time, it should be recognized normal fertility is possible in men whose sperm counts have been suppressed well into the oligospermic range (<5 million per ml) as a result of exogenous steroid administration (7).

**Figure 1:**
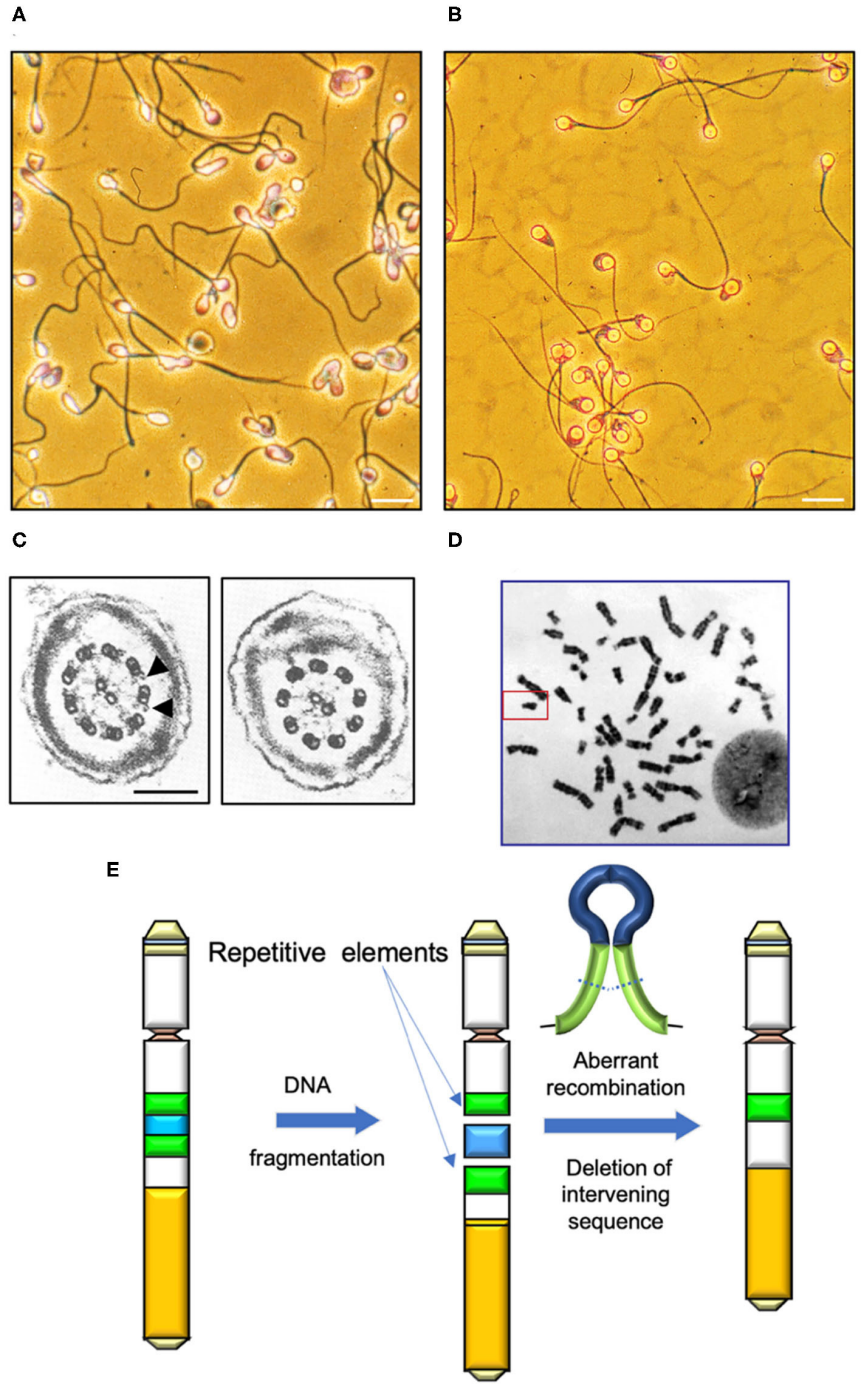
Determinants of human sperm quality. **(A)** Micrograph of a typical human sperm population. Note the significant inter-cell variation in morphological appearance of the sperm head, midpiece and tail. Morphological assessments are generally couched in terms of percentage normal cells, however defining the precise attributes of “normal” in this context is difficult. Scale bar = 10 μm. **(B)** A clearly abnormal condition is “globozoospermia” characterized by round headed, acrosome-less cells that are incapable of fertilization even though their motility is normal (10). Scale bar = 10 μm. **(C)** In contrast, Kartgener syndrome is a genetically-determined condition associated with major defects in the axoneme and complete immotility. Left panel cross section through a normal sperm tail highlighting the dynein arms (arrowed); right panel shows a section through the axoneme of a Kartagener syndrome patient, showing the complete absence of dynein arms (11). Scale bar = 200 nm **(D)** Human karyotype with the Y-chromosome, framed. **(E)** Major mechanisms by which DNA fragmentation on the Y is induced is through the aberrant recombination of repetitive elements (green) and deletion of the intervening DNA (blue).

We can conclude from such studies that the traditional approach to male infertility diagnosis, which relies on comparing each element of the semen profile with thresholds of normality established by the World Health Organization, is seriously flawed, no matter how carefully those thresholds were established (8). For the most part, male fertility is not a question of possessing more than a certain critical number of motile, morphologically normal spermatozoa in the ejaculate. It is not a binary phenomenon that allows us to classify patients as “fertile” or “infertile” groups. With the exception of patients exhibiting azoospermia or certain genetic defects such as Kartagener syndrome, most males are on a continuum of relative fertility. Where any given patient lies on this continuum cannot be reliably ascertained by conventional semenology. The diagnostic value of the conventional semen profile lies only in its ability to reflect the quality of the underlying spermatogenic process. Although there may be general low-level statistical correlations between morphology or total sperm number and fertility, the ranges for each of these criteria are so broad that their prognostic significance is very limited (9)–and they tell us nothing about etiology (Figure 1A). Terms such as asthenozoospermia, oligozoospermia, and teratozoospermia are convenient, descriptive terms. However, they are not diagnoses in the true sense of the word. As a consequence, we have greatly overestimated our understanding of the causes of human infertility and, in reality, have very limited insight into the overall contribution made by the “male factor.” Much of male infertility is, in fact, idiopathic.

Fortunately, this bleak landscape is gradually changing as we acquire more knowledge of the mechanisms regulating sperm production and function. For example, the impact of genetics and epigenetics on male fertility has been revealed in recent years, largely as a consequence of technical advances in our ability to screen the genome for differences in DNA methylation profile and single nucleotide polymorphisms/mutations. Similarly, many studies describe oxidative stress as another significant cause of male infertility that influences the functionality of spermatozoa and the integrity of their DNA. In this review, we shall consider the progress that has been made in establishing the relative contributions of genetics and oxidative stress to the etiology of previously unexplained male infertility. We shall also tentatively present a unifying hypothesis suggesting that these two contributors to the pathophysiology of male reproduction may well be causally linked.

## Genetic Causes of Male Infertility

### Mutations Affecting Sperm Structure and Function

Mutations and epimutations in the male germ line can affect the functional competence of spermatozoa and/or their primary production. Examples of mutations that influence sperm quality include conditions that affect the morphological appearance of spermatozoa and their competence for fertilization. A classic example of such a condition is globozoospermia. Patients exhibiting globozoospermia produce spermatozoa with spherical-shaped heads, no acrosome and a disorganized midpiece characterized by excess amounts of residual cytoplasm (Figure 1B). Interestingly, globozoospermic spermatozoa possess normal flagella that are capable of progressive motility and, under the appropriate conditions, can exhibit hyperactivation. However, these cells are incapable of binding to the zona pellucida and achieving sperm-oocyte fusion, even after treatment with the calcium ionophore, A23187 (10). These functional defects can be circumvented through the application of ICSI, although, even with this technique, fertilization rates are low. One of the reasons for the lack of fertilization is that globozoospermic spermatozoa are deficient in phospholipase C zeta (PLCζ), the calcium oscillogen that orchestrates oocyte activation at fertilization. Artificial oocyte activation in concert with ICSI can enhance fertilization rates with globozoospermic spermatozoa, but even with this intervention success rates are still significantly lower than control levels (12). One possible explanation for the poor fertilization rates observed with such spermatozoa is that they apparently exhibit high levels of DNA fragmentation in association with poor chromatin packaging and protamine deficiency (13). A related syndrome is “partial globozoospermia” where >50% of cells exhibit the round-headed acrosomeless morphology (14). Although the genetic basis of this partial condition remains uncertain at the present time, the presence of apparently normal cells in the ejaculate means that it can be very effectively treated with ICSI (15).

The causes of globozoospermia are genetic and, as far as we are currently aware, involve mutations in three genes, *SPATA16* (spermatogenesis associated 16), *PICK1* (protein interacting with PRKCA 1) and *DPY19L2* (DPY-19 like 2) with the latter being dominant (16). Homozygous deletions in *DPY19L2* are caused by non-allelic homologous recombination between flanking LCR (low copy repeat) sequences in around 70% of cases (17). Like most genetic conditions responsible for male infertility these causative mutations are rare (<1%). The major *DPY19L2* deletion is thought to have stabilized at a low level within the population because the *de novo* production of mutant alleles balances the negative selection incurred by sterile homozygous males (18).

A second sperm defect with a recognized genetic cause is macrozoospermia, characterized by the presence of a very high percentage of spermatozoa with enlarged heads and multiple flagella. A majority of such cells are diploid and the etiology involves mutations in the *AURKC* (aurora kinase C) gene (16). The incidence of these mutations varies from population to population but a recent analysis of North African males suggested that AURKC mutations were the most common, comprising 2.7% of the infertile male population compared with 1.2% exhibiting *DPY19L2*-dependent globozoospermia and anticipated rates of 1.6% exhibiting Klinefelter syndrome and 0.23% with Y-chromosome deletion (19). Macrozoospermia is observed in patients who are homozygous recessive for AURKC mutations. Two mutations in AURKC have been described: A p.Y248^*^ non-sense mutation which arose 925–1,325 years ago and the c.144delC homozygous frameshift mutation, dated as having occurred 250–650 years ago. In order to account for the retention of these mutations in the population over so many generations, it has been suggested that the heterozygote must have some selective reproductive advantage possibly related to sperm production (20); however, there is no evidence to support this suggestion at present.

Sperm head morphology is also radically altered in a condition known as acephalic spermatozoa. Mutations in several different genes are known to generate this phenotype. Thus, polyamine modulated factor 1 binding protein 1 (*PMFBP1*), testis specific 10 (*TSGA10*), Sad1 and UNC84 domain containing 5 (*SUN5*), bromodomain testis associated (*BRDT*) and centrosomal protein 112 (*CEP112*) have all recently been implicated in the etiology of this condition (21–23). The reason why so many different mutations appear to be involved in the creation of acephalic spermatozoa is a reflection of the inordinate complexity of the head-tail coupling apparatus in these cells (24). Mutations in *SUN5* account for about half of all cases of acephalic spermatozoa in human patients. In some ways this is fortunate, because patients with *SUN5* mutations can be successfully treated using ICSI (25), reflecting the very specific role this protein plays in anchoring the sperm head to the tail (26). However, other mutations associated with the acephalic syndrome such as *TSGA10* and *CEP112* are not readily treatable with assisted conception therapy because the damage is centered on the sperm centriole and, in humans, the paternal centrioles are responsible for orchestrating cell division in the embryo. In the absence of functional centrioles in the spermatozoa, fertilization may be achievable with ICSI but any embryo created will exhibit arrested embryonic development (22).

Just as the complexity of sperm head attachment means that the acephalic condition is potentially associated with mutations in any one of a number of key genes, exactly that same is true of defects in the flagellum in the etiology of asthenozoospermia. We have known about defects in the axoneme in the pathological suppression of sperm motility since the pioneering work of Afzelius (27, 28) recorded defects in several components of the axoneme of men exhibiting either a complete lack of sperm motility or severe asthenozoospermia. These diseases are grouped under the heading of primary ciliary dyskinesias (PCDs) because they affect all ciliary structures in the body, not just the sperm flagella. A classic example of this condition is Kartegener syndrome (Figure 1C), which is associated with a complete lack of sperm motility due to the absence of dynein arms, in association with chronic sinusitis, bronchiectasis and, in 50% of cases, situs inversus caused by an inability of the embryonic cilia to shift the heart to the left hand side. The condition is inherited in an autosomal recessive manner as a result of biallelic homozygous or compound heterozygous mutations in several candidate genes including coiled-coil domain containing 40 (*CCDC40*), dynein axonemal heavy chain 1, 5, and 11 (*DNAH1, DNAH5, DNAH7 DNAH11*) dynein axonemal intermediate chain 1 (*DNAI1*), leucine rich repeat containing 6 (*LRRC6*), Zinc finger MYND-type containing 10 (*ZMYND10*) armadillo repeat containing 4 (*ARMC4*) and tetratricopeptide repeat domain 12 (*TTC12*) (29–33). Overall, more than 40 genes have been implicated in this heterogenous disease to date and the list of genes involved is expanding rapidly in concert with improvements in our understanding of ciliary structure and function. In theory, any gene involved in the assembly, structure and function of ciliary/flagellar structures could contribute to the male infertility associated with PCD. The condition is rare (prevalence 1:10,000 to 1:40,000 births) and the ultrastructural phenotypes are variable involving no outer and inner dynein arms (DAs), outer DAs alone, inner DAs with microtubular disorganization or defects yielding an abnormal central complex. Because of this heterogeneity, not all patients exhibiting PCD are infertile. Importantly apart from the loss of motility, spermatozoa obtained from Kartagener syndrome have been shown to be functionally normal in that they will engage in the process of capacitation, will acrosome react and, if physically manipulated to lie close to the plasma membrane of the oocyte will achieve sperm-oocyte fusion (11). So, even if motility loss is total, conceptions can still be achieved using either sub-zonal insemination (SUZI) techniques that place immotile, but acrosome reacted, spermatozoa adjacent to the vitelline membrane of the oocyte or ICSI (34). Outside of such assisted conception procedures, spontaneous pregnancies are possible with PCD patients providing the loss of motility is partial; however, spontaneous fertility is unlikely with mutations in certain genes including *CCDC39, CCDC40*, dynein axonemal assembly factor 1 (*DNAAF1)* and *LRRC6* (35).

In addition to PCD, where multiple ciliopathies are observed in different organ systems, male infertility is also associated with sperm motility defects involving genes that specifically impact the development of the sperm tail, its detailed architecture and its physiological regulation. These mutations generate isolated male infertility associated with severe asthenozoospermia in the absence of any other pathology and are known collectively as Multiple Morphological Abnormalities of the sperm Flagella (MMAF). The list of mutations responsible for MMAF is expanding rapidly but currently includes mutations in adenylate kinase 7 (*AK7*) (36), glutamine rich 2 (*QRICH2)* (37), cilia and flagella associated proteins (*CFAP43, CFAP44, CFAP65, CFAP69, CFAP70, CFAP91, CFAP251*) (38–44) WD repeat domain 19 (*WDR19*) (45), DAZ interacting zinc finger protein (*DZIP1*) (46), *DNAH1* (also implicated in PCD) (47) *DNAH2* (48), *DNAH6* (49), *DNAH17* (50), *TTC29* (51), *TTC21A* (52), armadillo repeat containing 2 (*ARMC2*) (53) *CEP135* (centrosomal protein 135) (54). fibrous sheath interacting protein 2 (*FSIP2*) (55), ADP ribosylation factor like GTPase 2 binding protein (*ARL2BP*) (56), sperm flagellar 2 (*SPEF2*) (57), and DnaJ heat shock protein family (*Hsp40*) member B13 (*DNAJB13*) (58).

Of course, motility is not the only attribute of sperm function susceptible to interference by genetic and epigenetic mutations. For example, we know that genetic defects in sperm PLCζ, which activates the generation of calcium transients in the fertilized oocyte, impair human oocyte activation and fertilization (59). Fertilization failure has also been associated with mutations in *CATPSERE* (CatSper-epsilon), a component of the sperm calcium channel (60) as well as polymorphisms in the mitochondrial genes MT-ATP6 and MT-CYB (61).

It must be evident from the information presented above that there are a great number of mutations potentially capable of suppressing the fertilizing potential of human spermatozoa. This may not be surprising given the biochemical sophistication underpinning such complex sperm functions as sperm motility (which accommodates a variety of sophisticated behaviors including rheotaxis, chemotaxis and thermotaxis as well as the switch to hyperactivation), sperm transport to the site of fertilization, the ability of a capacitated spermatozoon to recognize just one other cell type in the body (the egg), the induction of acrosomal exocytosis, sperm-oocyte fusion and oocyte activation. Moreover, these are just the mutations affecting the structure and function of spermatozoa, there are a great many more mutations influencing the primary production of spermatozoa at a testicular level.

### Mutations Affecting the Testes and Excurrent Ducts

Obstructive azoospermia is present in around 30% of azoospermia cases and is often due to occlusion of the excurrent duct system subsequent to infection, trauma or surgery. The major genetic cause involves CFTR (Cystic fibrosis transmembrane conductance regulator) mutations that are known to induce abnormal formation or bilateral absence of the vas deferens (62). The remaining 70% of azoospermia cases are non-obstructive and relate to primary failure of spermatogenesis. Primary testicular failure may be observed with different forms of testicular cancer, the genetic origins of which are as complex as their histopathology, including germ cell tumors (seminoma, embryonal carcinoma, yolk sac tumor, and teratoma) and, in older men, testicular lymphomas (63, 64). Gains of chromosome arm 12p and aneuploidy are nearly universal in germ cell tumors (65) while primary testicular lymphomas involve near-uniform loss of CDKN2A (cyclin dependent kinase inhibitor 2A) with rare TP53 (tumor protein p53) mutations as well as 9p24.1/PD-L1/PD-L2 copy number alterations and additional translocations of these loci (66).

Cancer aside, an increasing list of monogenic gene mutations are being associated with non-obstructive azoospermia. Just as we saw with mutations affecting sperm structure and function, gene mutations leading to primary testicular failure are many, varied and infrequent, reflecting the underlying complexity of the spermatogenic process and the inability of any particular mutation to become anything other than rare, given: (1) the negative selection pressure associated with male infertility, (2) the fact that many of these mutations also cause infertility in women and so cannot find refuge in the female germ line (67), and (3) the absence of any particular reproductive advantage in the heterozygous form. These mutations are generally autosomal recessive and inherited from fertile parents in homozygous, compound heterozygous or hemizygous form. However, autosomal dominant monogenic mutations can also precipitate a state of infertility including *SYCP3* (Synaptonemal complex protein 3), *NR5A1* (nuclear receptor subfamily 5 group A member 1) and the WT1 (Wilms' tumor 1) gene (68). A recent detailed analysis of the genetic causes of non-obstructive azoospermia (NOA) concluded that the largest single category of monogenic defects detected in NOA patients comprises genes involved in different stages of spermatogenesis, mostly functioning in the prophase of the first meiotic division as well as transcriptional and endocrine regulators of reproduction (67).

Of course, not all mutations causing non-obstructive azoospermia are expressed in the testes. There are several genes involved in the etiology of hypogonadotrophic hypogonadism that are key components of the hypothalamic-pituitary-gonadal axis involved in the endocrine regulation of spermatogenesis. The human *GNRHR* (gonadotropin-releasing hormone receptor) gene is a case in point. This protein is a G-protein coupled receptor expressed on the surface of pituitary gonadotrophs that respond to pulsatile GnRH stimulation by promoting the secretion of gonadotrophins FSH and LH. Several (at least 19) mutations have been identified in this gene that often exert their pathological action as compound heterozygotes (68). There are also mutations that interfere with the differentiation of the male reproductive system including cytochrome b5 type A (*CYB5A*) which selectively disrupts 17,20-lyase activity leading to disordered sexual development (69) and deletions on chromosome 21 that also cause defects in male sexual development (70). Mutations on the X chromosome are also linked with male infertility including: anosmin 1 (*ANOS1*), a gene linked to Kallmann syndrome (71), testis expressed 11 (*TEX11*), linked to meiotic arrest (72) and nuclear receptor subfamily 0 group B member 1 (NR0B1) associated with adrenal hyperplasia and hypogonadotropic hypogonadism (73). Mutations in the X-linked androgen receptor gene are also known to induce infertility in around 2% of male patients (74).

Overall, the foregoing summary reveals a bewildering array of monogenic defects involved in male infertility exerting their action at all stages of gonadal development and function, from the initial morphogenesis of the male genital tract in the fetus to the differentiation and maturation of fully functional spermatozoa. However, interestingly, the most common genetic causes of male infertility are not single gene mutations at all, but aneuploidies of which Klinefelter syndrome (XXY) is the most common, accounting for around 10% of patients with non-obstructive azoospermia/severe oligozoospermia (75, 76).

In view of the strong negative selection pressure associated with infertility, the maintenance of such an impressive range of infertility-inducing chromosomal or genetic mutations in the general population must involve the steady spontaneous generation of *de novo* lesions affecting genes and chromosomes involved in the spermatogenic process (77). The importance of such *de novo* mutations in the etiology of male infertility is beautifully illustrated by the presence of Y-chromosome deletions in males exhibiting spontaneous severe oligozoospermia on non-obstructive azoospermia.

## The Y Chromosome

The Y chromosome is unusual because it lives in isolation (Figure 1D). One detrimental consequence of such a solitary existence is that the Y chromosome has limited options when it comes to the repair of DNA damage. All other chromosomes have a homologous chromosome, inherited from the other parent, that can assist in the repair of damage DNA via homologous recombination. The Y chromosome has no homolog to recombine with and so it has reverted to an intrachromosomal form of recombination in order to maintain a modicum of stability. Typically, genes on the Y-chromosome make multiple copies of themselves as a buffer against the chaos that would be introduced by the accumulation of deleterious spontaneous mutations, a lack of selection pressure, the presence of unwanted genetic hitchhikers and genetic drift. Some of these repetitive elements have become inverted to create palindromic sequences that facilitate recombination events in an area of the genome where inter-chromosomal recombination is otherwise suppressed. As a consequence of this strategy, damage to key genes on the Y-chromosome can be repaired by the affected gene recombining with a palindromic copy of itself in a process known as gene conversion. This capacity for intra-chromosomal recombination has enabled the Y chromosome to stabilize after an initial period of exponential decay such that no genes have been lost since the divergence of humans and chimpanzees between 6 and 7 million years ago while only one gene had been lost since humans diverged from the rhesus macaque 25 million years ago (78, 79).

Unfortunately, the presence of such palindromic sequences, as well as repetitive retroviral elements on the human Y chromosome, facilitates the creation of chromosome deletions as a consequence of aberrant recombination events (Figure 1E). Three common Yq deletions that recur in infertile males are termed AZF (Azoospermia Factor) microdeletions–AZFa, AZFb and AZFc. In addition, the combinations, AZFbc, AZFabc, and a partial AZFc, called AZFc/gr/gr are also observed (80, 81). AZFa and AZFb deletions usually result in complete azoospermia, with no current potential for treatment. However, patients with AZFc deletions are typically characterized by severe oligozoospermia, with small numbers of spermatozoa recoverable from the ejaculate or from testicular biopsy material. These cells are sufficiently normal to permit treatment options involving ICSI. Of course, an inevitable consequence of the Y-chromosome's genetic isolation, is that any son generated as a consequence of ICSI will inherit his father's microdeletion and, thus, his infertility (80).

The prevalence of Y chromosome deletions and microdeletions is estimated at 1:2,000 to 1:3,000 males while the frequency of Yq microdeletions in males with azoospermia is 15% and with severe oligozoospermia about 5% (82). Unlike the monogenic gene mutations responsible for defective sperm production or primary testicular failure reviewed above, Y-chromosome mutations cannot be maintained in the population as heterozygotes or via passage through the female germ line. With very few exceptions, every new case of Y-chromosome deletion has been spontaneously created in the fertile father's germ line. This tells us that DNA damage and repair must be a major feature of male reproduction. Indeed, a recent analysis has found that 75% of all *de novo* mutations arise in the male germ line via mechanisms that have little to do with replication error, as commonly supposed (83). Rather, the high rate of mutations observed in the male germ line, including common C-to-G transversions and CpG transitions show genomic distributions and sex-specific age dependencies indicative of double-strand break repair and methylation-associated damage, respectively (83). The Y-chromosome's strategy of gene amplification and intra-chromosomal recombination in order to stabilize has, undeniably, been effective in slowing the rate of gene attrition on this chromosome. However, the presence of so many repetitive palindromic elements also creates a measure of vulnerability. If DNA fragmentation rates are high, then there is the potential for distant palindromic sequences to recombine, resulting in deletion of the intervening genetic information. Such deletions could either occur during spermatogenesis or, in principle, in the oocyte as a result of aberrant DNA repair prior to the S-phase that precedes the first cleavage division. Thus, Y-chromosome deletions, like many mutations that arise in our species may be seen as a consequence of the high rates of DNA damage and fragmentation that characterize the male germline. In this context, there is a significant body of evidence indicating that DNA fragmentation is a consistent feature of human spermatozoa and that the induction of such damage is oxidative.

## Oxidative Stress

The importance of oxidative stress in the etiology of defective sperm function has been recognized since the pioneering studies of Thaddeus Mann and colleagues at the University of Cambridge demonstrated that mammalian spermatozoa were vulnerable to a lipid peroxidation process that attacks the unsaturated fatty acids in these cells, destroying the plasma membrane and compromising their functional competence (84). The induction of such stress may involve the enhanced generation of reactive oxygen species (ROS) by these cells and/or a deficiency in the levels of antioxidant protection they are afforded (85–87). The net impacts of oxidative stress include a loss of motility, a decrease in the ability of the spermatozoa to undergo the acrosome reaction, an impaired capacity to fuse with the vitelline membrane of the oocyte—and DNA damage (85, 88–90).

### Leukocyte Infiltration and ROS Generation

**Figure 2:**
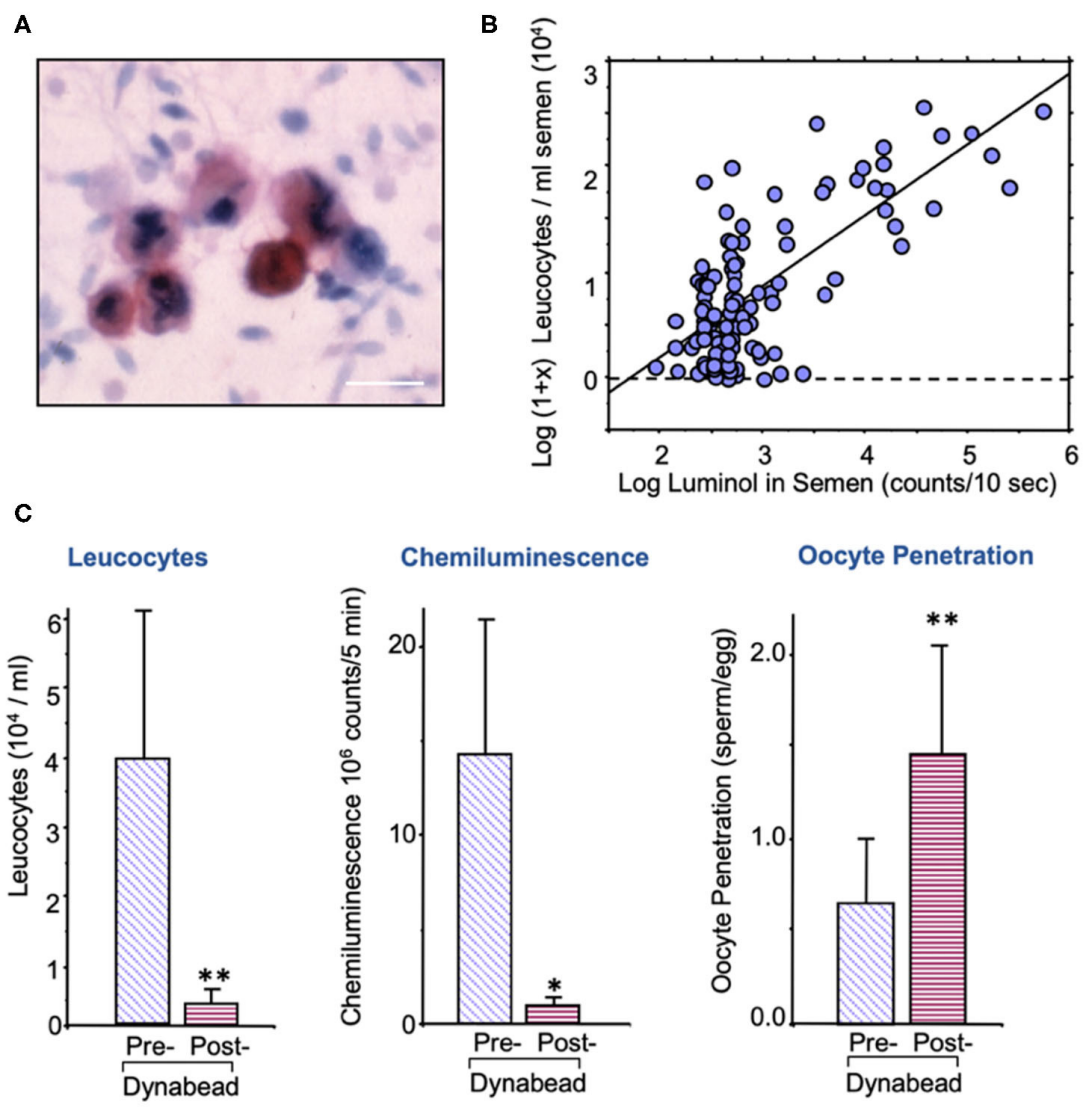
Leukocyte contamination of human semen samples. **(A)** All human semen samples are contaminated with leukocytes, largely neutrophils, and macrophages; sample stained with an antibody against the common leukocyte antigen, CD45. Sale bar = 10 μm. **(B)** The spontaneous generation of reactive oxygen species by human semen samples is highly correlated with the level of leukocyte contamination. **(C)** Treatment of washed human sperm suspensions with magnetic beads coated in anti-DC45 removes a majority of the leukocytes, reduces the levels of oxidative stress as determined by luminol dependent chemiluminescence and increases fertilization rate in the heterologous sperm-oocyte fusion assay (91). *P < 0.05; **P < 0.01.

The sources of ROS that create this oxidative stress are complex and may be due to either intrinsic or extrinsic factors. The major extrinsic factor are leukocytes that enter the semen at the moment of ejaculation from the secondary sexual glands. The major leukocyte species in this context are neutrophils (Figure 2A) that arrive in the seminal compartment in an activated, free radical-generating state (Figure 2B). The presence of these cells is thought to reflect an underlying reproductive tract infection (92, 93), although other factors such as trauma, surgery, and autoimmunity could also be involved. As long as leukocyte numbers are relatively low (less than the leukocytospermic threshold of 1 million/ml), the ROS generated by infiltrating leukocytes have no effect on the functionality of the spermatozoa because these cells are adequately protected by the powerful antioxidants present in seminal plasma (94). However, if the leukocyte numbers exceed this leukocytospermic threshold, or are just below it, then a state of oxidative stress prevails, and sperm function is compromised (95). Furthermore, if leukocytes are still present in the washed sperm suspensions used in assisted conception procedures then significant oxidative stress will again be created due to the absence of significant antioxidant protection in conventional IVF culture media. The existence of low-level leukocyte contamination in washed human sperm suspensions is a significant issue for IVF therapy because it negatively impacts the fertilization rates subsequently observed (96, 97). This problem can either be addressed through the incorporation of antioxidants such as N-acetylcysteine or hypotaurine in the culture medium (98) and/or through the selective removal of contaminating leukocytes using magnetic particles coated with a monoclonal antibody against the common leukocyte antigen (Figure 2C) (91). Such treatments effectively reduce levels of oxidative stress in the sperm suspensions and significantly enhance the fertilization rates subsequently observed.

### Spermatozoa and ROS Generation

Outside of leukocyte contamination, a majority of the ROS that compromise sperm function are generated endogenously via a variety of pathways, in response to a variety of stimuli. It is important to point out that the levels of ROS generated by spermatozoa are orders of magnitude lower than leukocytes (99). This is the reason why flow cytometry is such a useful technique for monitoring seminal ROS production because it enables the separation of spermatozoa from other cell types and simplifies interpretation of the data. By contrast, techniques such as luminometry, always run the risk of generating data that is heavily influenced by the presence of contaminating leukocytes (100, 101). It should also be recognized that a vast majority of the probes used with flow cytometry are redox active agents that do not measure ROS directly but rather, oxidative activity. Nevertheless, by using dihydroethidium as a probe and separating out the reaction product specifically generated by superoxide anion, 2-hydroxyethidium, it has been possible to generate evidence of superoxide production by mammalian spermatozoa (102, 103). Moreover, these data have been confirmed with definitive techniques such as electron paramagnetic resonance spectroscopy (104–106).

There has also been discussion as to which ROS is the more important in the determination of sperm function, superoxide (107) hydrogen peroxide (86, 108–110) nitric oxide (111) or peroxynitrite (112). In reality, all these oxidants and free radical species are so reactive that they are constantly interconverting and contributing to the oxidative stress experienced by the male gamete. It is doubtful whether any particular species actually pre-dominates.

### Sperm Mitochondria as a Source of ROS

**Figure 3:**
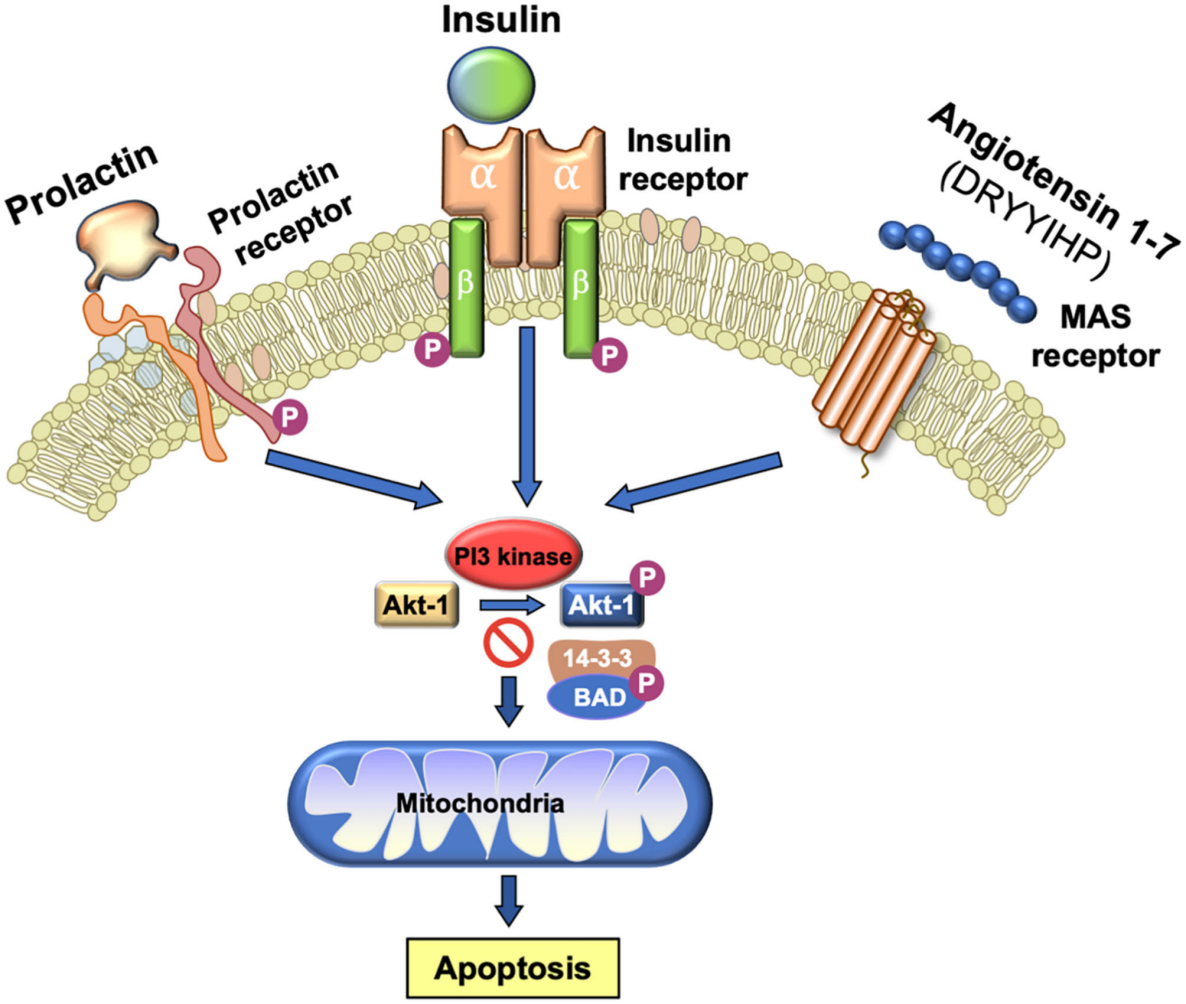
Importance of PI3 kinase in the maintenance of sperm viability. As long as PI3 kinase is phosphorylated, its downstream target AKT1 is also phosphorylated. Phosphorylated AKT1 in turn phosphorylates BAD which, in this state, remains bound to its 14-3-3 keeper protein. This system is driven by a series of prosurvival factors such as prolactin, insulin and angiotensin 1–7 that act through their cognate receptors to maintain PI3 kinase in a phosphorylated state. If this system is perturbed and PI3 kinase becomes dephosphorylated, then BAD reverts to its dephosphorylated state, releases 14-3-3 and moves to the mitochondria where it forms heterodimers with Bcl-2 and Bcl-xL, inactivating these regulators and allowing Bax/Bak-triggered apoptosis. The induction of PI3 kinase phosphorylation is therefore essential for sperm survival.

One of the major sources of superoxide anion within spermatozoa are the mitochondria (113). These organelles generate ROS as a normal by-product of aerobic metabolism due to the leakage of electrons from the mitochondrial electron transport chain, which are then swept up by the universal electron acceptor, oxygen, to generate superoxide anion. Mitochondrial ROS are also produced as part of the intrinsic apoptotic cascade that becomes activated whenever the phosphoinositide signaling pathway is compromised (114). Under physiological circumstances, a variety of pro-survival factors, including as insulin, prolactin or angiotensin 1–7 (115, 116) stimulate phosphorylation and activation of phosphoinositide-3 kinase (PI3K). The latter in turn phosphorylates another kinase, AKT. As long as AKT is phosphorylated, downstream targets of this kinase such as the apoptosis regulator, BCL2-associated-agonist-of-cell-death (BAD) are also phosphorylated. Phospho-BAD forms a heterodimer with its 14-3-3 keeper protein, leaving Bcl-2 free to inhibit Bax-triggered apoptosis, thereby maintaining spermatozoa a viable motile state (114). However, if PI3K activity is disrupted, AKT and its downstream target, BAD, become dephosphorylated allowing the latter to escape from the grip of its 14-3-3 keeper to form a heterodimer with Bcl-2 and Bcl-xL, inactivating these regulators and thus allowing Bax/Bak-triggered apoptosis. The intrinsic apoptotic cascade is associated with rapid motility loss, mitochondrial ROS generation, caspase activation in the cytosol, annexin V binding to the cell surface, cytoplasmic vacuolization and oxidative DNA damage (Figure 3) (114). So, any stress factor that will induce an apoptotic response in human spermatozoa will trigger mitochondrial ROS generation and a loss of sperm function.

Mitochondrial ROS generation and apoptosis may also be important in the mechanisms underpinning sperm senescence. All mammalian spermatozoa have a finite life span and after a few days (depending on species) will become senescent *in vivo* and *in vitro*, losing their vitality, motility, tyrosine phosphorylation status and DNA integrity with the passage of time (117, 118). Oxidative stress appears to be one component of the senescence process judging from the fact that sperm motility and DNA integrity can be significantly improved *in vitro* if oxygen tensions are reduced and/or antioxidants are incorporated into the medium (119–121). Furthermore, the generation of ROS on prolonged incubation has been found to increase with time and a majority of this ROS appears to be mitochondrial, possibly reflecting the progressive entry of senescent cells into the intrinsic apoptotic pathway (121, 122). Accordingly, antioxidants that target mitochondrial ROS generation such as co-enzyme Q10 and pyrroloquinoline quinone as well as thiols such as N-acetylcysteine and penicillamine have been found to extend sperm motility *in vitro* (121, 123, 124). Similarly, treatments that divert energy generation away from mitochondrial oxidative phosphorylation and toward glycolysis, such as exposure to rosiglitazone, decrease mitochondrial ROS generation, and allow spermatozoa to maintain high levels of motility *in vitro* for at least 6 days (125). However, there is still some conjecture as to how many other factors are involved in sperm motility loss *in vitro*. In a recent study of human spermatozoa incubated over a 5 day period, the loss of virtually all motility was not accompanied by a corresponding increase in 4-HNE levels by Western blot (102). In light of these data, we have to conclude that there may be multiple reasons why spermatozoa become senescent *in vitro* and we are yet to resolve the full complexities of this process.

Mitochondrial ROS can also be triggered by a range of amphiphilic compounds in human spermatozoa including cis-unsaturated fatty acids; the polar nature of these compounds favoring their corporation into mitochondrial membranes, altering membrane fluidity and facilitating electron leakage (126). Since the free unsaturated fatty acid content of defective human sperm populations is positively correlated with the induction of mitochondrial superoxide generation, we can conclude that the pathophysiology of defective sperm function is at least partly dependent on changes to the lipid composition of these cells (127). Mitochondrial ROS can also be stimulated by toxicants that can perturb the flow of electrons along the mitochondrial electron transport chain. For example, the common preservative, parabens (a mixture of parabenzoic esters), has been shown to stimulate mitochondrial ROS in spermatozoa in a manner which is correlated with alkyl chain length (128). Similarly, the xenoestrogen bisphenol A stimulates mitochondrial ROS generation by human sperm mitochondria (129) as do certain polyphenols (epigallocatechin gallate, genistein, didox gossypol) several of which are traditionally regarded as antioxidants (130).

The cryopreservation of spermatozoa is another situation in which sperm function is compromised partly as a consequence of oxidative stress created by enhanced mitochondrial ROS generation. Consequently, a large number of studies, conducted in a range of different species, have examined the impact of antioxidants on post-thaw functionality. Since antioxidants such as L-carnitine and Mito Tempo have already proven effective in this regard (131, 132), a systematic comparison of antioxidants targeting the mitochondria is now warranted, to determine the optimal formulation for protecting spermatozoa against cryostorage injury.

Another extraneous factor which is thought to enhance mitochondrial ROS generation in mammalian spermatozoa is radiofrequency electromagnetic radiation (RFEMR) (133). The concept that RFMR induces electron leakage from the mitochondrial electron transport chain, and thus promotes superoxide anion generation (134), is controversial but has received support from numerous independent studies (135). Mitochondrial ROS generation activated by RFEMR has, in turn, been associated with the suppression of sperm motility, the induction of apoptosis, the loss of local antioxidant protection and the stimulation of sperm oxidative DNA damage both *in vivo* and *in vitro* (136–140).

*In vitro* exposure to electromagnetic radiation in the form of UVB light will also trigger mitochondrial ROS generation in human spermatozoa (141) as will the *in vitro* exposure of these cells to temperatures above 40°C (134). Mild heat stress (35°C) has also been found to activate mitochondrial ROS generation *in vivo*, with round spermatids being particularly vulnerable to this form of stress (135).

Mitochondrial ROS generation can also be stimulated by the electrophilic lipid aldehydes generated as an end-product of lipid peroxidation. Not all lipid aldehydes are equivalent in this respect, the differences generally correlating with the second order rate constants describing their interaction with the model nucleophile, glutathione. Acrolein and 4-hydroxynonenal (4-HNE) are the most active, stimulating mitochondrial ROS generation by binding to components of the mitochondrial electron transport chain, particularly succinic acid dehydrogenase (142, 143). The capacity of lipid aldehydes generated as a result of oxidative stress to bind to components of the mitochondrial electron transport chain and stimulate yet more ROS generation means that once this process is initiated, it becomes a self-perpetuating process unless a chain breaking antioxidant intervenes.

### NAD(P)H Oxidase

**Figure 4:**
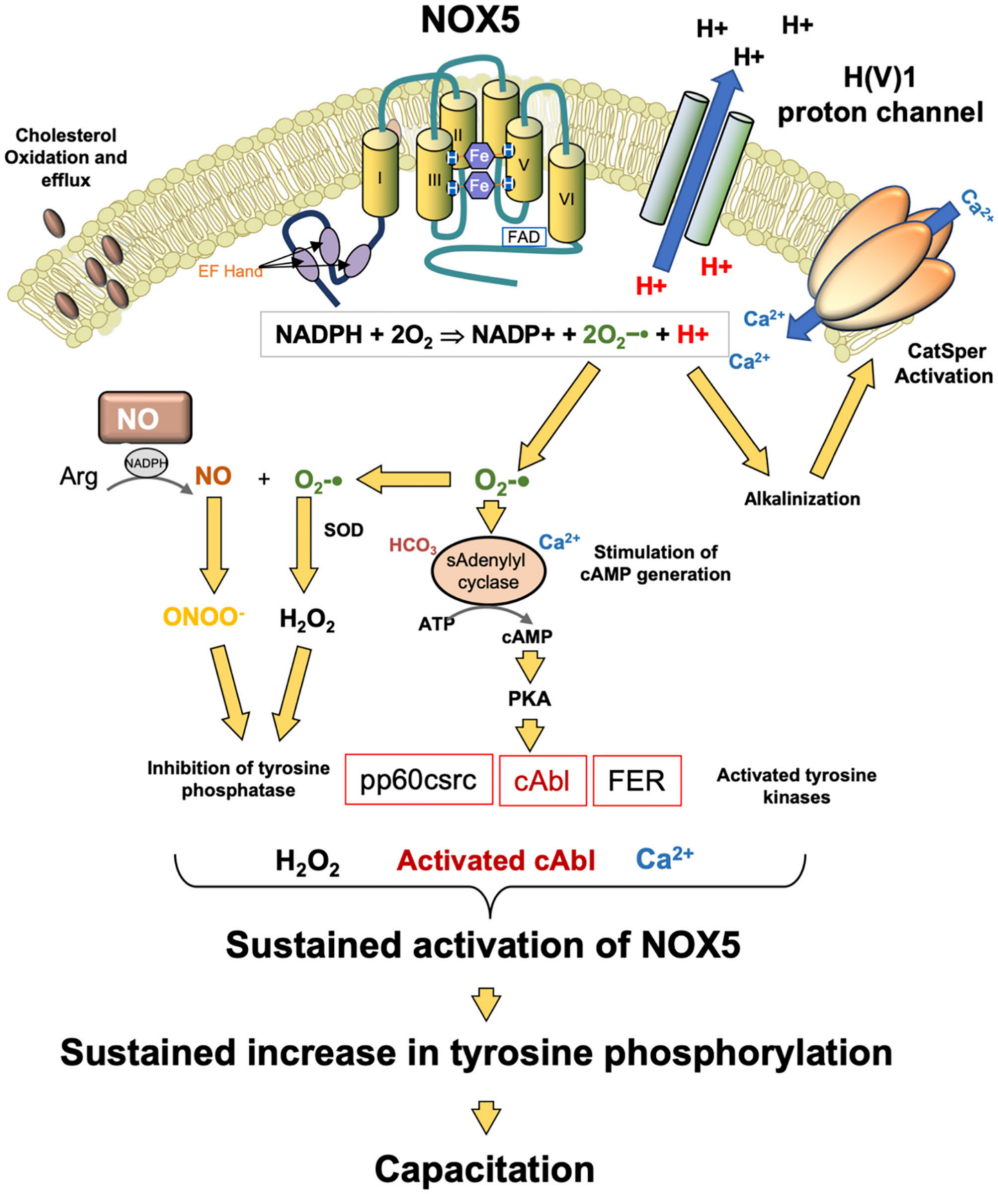
Potential role for NOX5 in the regulation of sperm capacitation. NOX5 catalyzes the generation of superoxide anion and protons. The latter activate the H(V)1 proton channel leading to proton extrusion and alkalinization of the cytoplasm. This pH change activates the CatSperm channel leading to calcium influx which in turn further promotes NOX5 activity. The other product of NOX5 action, superoxide anion participates tin the activation of soluble adenylyl cyclase and the generation of cAMP. cAMP activates PKA which then phosphorylates as number of tyrosine kinases. At the same time, hydrogen peroxide and peroxynitrite (ONOO^−^) co-operate to silence tyrosine phosphatase activity leading to a global increased in phosphotyrosie expression. One of the kinases activated in this process, cAbl, as well as hydrogen peroxide, are both known to stimulate NOX5 activity. As a consequence of these activities, NOX5 is maintained in an activated state, driving the tyrosine phosphorylation cascades that culminate in the attainment of a capacitated state.

Another potential source of ROS generation in human spermatozoa are NAD(P)H oxidases reminiscent of the enzyme responsible for the oxidative burst in phagocytic leukocytes. Several authors (144, 145) have suggested that spermatozoa generate ROS via such enzymes based on the inhibitory action of diphenylene iodonium (DPI). However, DPI is a generalized flavoprotein inhibitor and so also suppresses ROS generation by oxidoreductases in the mitochondrial electron transport chain. This, and other factors, has, in some minds, cast doubt on the existence of such an enzyme in human spermatozoa and its possible contribution to oxidative stress in the male germ line (146, 147). However, in 2002, Banfi et al. (148) described the existence of an NADPH oxidase (NOX5) in several cell types, including human spermatozoa. Musset et al. (149) subsequently confirmed immunocytochemically that this oxidase was not only present in the neck or acrosomal region of these cells but generated superoxide anion in a calcium-dependent manner. They also identified cAbl as a tyrosine kinase associated with the activation of this oxidase. In addition, exposure to hydrogen peroxide was found to activate ROS generation by NOX5. These findings explain why several authors (150–152) have found that sustained ROS generation by human spermatozoa can be triggered by transient exposure to an oxidizing agent such as hydrogen peroxide—it also provides a mechanism. ROS generation is known to be associated with the stimulation of tyrosine phosphorylation in mammalian spermatozoa through the suppression of tyrosine phosphatase activity and the activation of adenylyl cyclase (153–155). cAMP is, in turn, known to activate cAbl in spermatozoa (156), and this kinase would then be expected to activate NOX5 leading to yet more ROS generation in concert with sperm capacitation, which is also cAMP driven (Figure 4).

The clinical significance of NOX5 is indicated by its high level of expression in the spermatozoa of asthenozoospermic males in concert with increases in superoxide and hydrogen peroxide generation and DNA damage (157). NOX5 expression has also been shown to be elevated in cases of teratozoospermia (158). Physiologically, the H(V)1 proton channel, which has been implicated in the regulation of sperm motility, is required for optimal superoxide production by spermatozoa via NOX5 (149), presumably by preventing cytoplasmic acidification that inevitably follows NADPH oxidation:

$$\begin{array}{l} \text{NADPH }+\;2{\text{O}}_{2}\Rightarrow \text{NAD}{\text{P}}^{+}\;+\;2{{\text{O}}_{2}}^{-•}\;+\;{\text{H}}^{+}\\ \text{ NADPH oxidase superoxide proton} \end{array}$$

Moreover, there is evidence that NOX5 and H(V)1 are involved in the induction of calcium signaling via Catsper in response to progesterone stimulation (159), again reflecting the fact that the activity of this calcium channel is highly sensitive to changes in pH (160) and that NOX 5 achieves intracellular alkalinization by activating the H(V)1 proton channel. Thus, it is possible that that NOX5 is a key regulator of sperm function as well as a potential mediator of oxidative stress and sperm pathology (Figure 4). However, we do not yet know why NOX5 activity would be elevated in the spermatozoa of infertile males. It is possible that the supply of NADPH is rate limiting in this situation and that cells possessing a large amount of residual cytoplasm (a characteristic feature of defective human spermatozoa suffering from oxidative stress) could fuel oxidase activity because they are over-endowed with glucose-6-phosphate dehydrogenase, a key regulator of the hexose monophosphate shunt responsible for regulating NADPH generation (161). We also do not understand how mouse spermatozoa can exhibit the same relationship between cAMP, ROS generation and sperm capacitation (162) but do not possess NOX5—possibly other oxidases are active in this species.

### Lipoxygenase

Another factor in the oxidative stress equation that may be upregulated in spermatozoa possessing excess residual cytoplasm is lipoxygenase. This enzyme is present in cytoplasmic droplets of defective spermatozoa (163) and catalyzes the dioxygenation of polyunsaturated fatty acids to the corresponding hydroperoxide. The enzyme facilitates the initial hydrogen abstraction step in the peroxidation process, creating a lipid radical that then combines with molecular oxygen to create a peroxyl radical that on protonation generates the corresponding hydroperoxide. There are two ways in which this chemistry could be associated with oxidative stress. The first is that in the presence of NADPH, lipoxygenases can generate superoxide anion directly (164). Pharmacological inhibition of the lipoxygenase, ALOX15, in precursor male germ cells and spermatozoa results in a significant reduction in both mitochondrial and cytoplasmic ROS generation, as well as a dramatic reduction in 4-HNE accumulation (165, 166). These findings are consistent with the ability of polyunsaturated fatty acids to stimulate ROS generation in human spermatozoa and thereby compromise sperm function (126). The second means by which excess lipoxygenase activity could create oxidative stress in these cells is by fueling the generation of lipid hydroperoxides in the sperm plasma membrane. These peroxides have the potential to initiate a lipid peroxidation cascade if sufficient transition metals (e.g., iron, copper) are present in the immediate vicinity (167). If left unbroken, such peroxidative chain reactions culminate in the generation of electrophilic lipid aldehydes such as 4-HNE which can have a devastating impact on sperm function by binding to key proteins involved in the execution of sperm function. In this context, incubating human spermatozoa in the presence of a lipoxygenase inhibitor has been shown to both reduce the levels of 4-HNE accumulation in human spermatozoa and promote the functional competence of these cells (166).

### L-amino Acid Oxidase

The first enzyme that was ever shown to generate ROS in mammalian spermatozoa was an L-amino acid oxidase. In their landmark papers published more than 70 years ago, Tosic and Walton (168, 169) described the presence of an enzyme in bovine spermatozoa that generated significant quantities of hydrogen peroxide using aromatic acids, such as phenylalanine. Interestingly dead cells were more responsive to phenylalanine stimulation because the disrupted plasma membrane allowed this amino acid substrate access to the oxidase. So, when bovine spermatozoa were exposed to high concentrations of aromatic amino acids (as happens when these cells are suspended in cryostorage media supplemented with egg yolk) the dead cells generated high amounts of hydrogen peroxide which then impeded the motility and fertilizing potential of live cells in the immediate vicinity. The same oxidase has been detected in equine (170), ovine (171), and human spermatozoa (172). In the latter, oxidase activity was lost from non-viable cells, so the “dead cell-influencing-live cell” scenario established for ungulate spermatozoa does not apply in the human situation. The physiological purpose of the oxidase is currently unclear, although in the presence of phenylalanine human spermatozoa develop many of the hallmarks of capacitation, including increased tyrosine phosphorylation and enhanced acrosome rates, via mechanisms that could be reversed by the concomitant presence of catalase. It is therefore possible that this enzyme contributes to the redox regulation of capacitation as spermatozoa ascend the female reproductive tract (172)-always assuming the bioavailability of adequate quantities of aromatic amino acids in this location.

### Antioxidant Protection

Spermatozoa possess very little cytoplasm and, as a consequence, they are deficient in the antioxidant enzymes that protect most cells from oxidative stress. This does not mean that these cells are totally lacking in any form of intracellular protection because they do possess a number of important antioxidant enzymes such as peroxiredoxin 6, superoxide dismutase, the glutathione peroxidase-reductase couple and limited catalase (86, 173–175). However, the level of protection afforded by these systems has finite limits with the result that spermatozoa are heavily dependent on extracellular antioxidants present the seminiferous tubule fluid (176) epididymal plasma (177), seminal plasma (178) and uterotubal fluid (179) to provide them with complete protection during their voyage from the seminiferous tubules to the vitelline membrane of the oocyte. As a result, any factors that impact the overall antioxidant status of an individual, and thus the bioavailability of extracellular antioxidants, can have an impact on the levels of oxidative stress suffered by the male germ line and thence fertility (180, 181). Examples of such conditions include diet (182), varicocele (183) smoking (184), obesity (185), heat stress (186), and environmental toxicants such as bisphenol A (187) all of which can impact systemic antioxidant tone and thus the vulnerability of spermatozoa to oxidative stress. Inadequate intracellular and extracellular antioxidant protection could therefore be a major factor in the etiology of both male infertility and oxidative DNA damage to the paternal genome (188). Given this background it would seem axiomatic that if oxidative stress is such a powerful cause of reproductive dysfunction in the male, then antioxidants should be part of the cure (189, 190). Unfortunately, the clinical trials needed to demonstrate that the administration of antioxidants to patients suffering demonstrable oxidative stress in their germ line will reap a therapeutic reward, have not yet been conducted at scale. Some promising pilot studies have been conducted with positive results (191) and efficacy has clearly been demonstrated in animal models (190). However, the gold standard randomized, double-blind, cross-over, placebo-controlled trial has yet to be conducted, partly because there is currently no consensus over the oxidative stress markers that should be used to identify appropriate patients for antioxidant therapy.

### Other Therapeutic Interventions

In addition to antioxidant administration we should also recognize that a variety of lifestyle interventions might also influence the oxidative stress underpinning male infertility including, increased exercise, improved diet as well as the cessation of alcohol consumption and cigarette smoking. Interestingly, the practice of yoga has been found to have a positive impact on the mRNA profile of human spermatozoa, their epigenetic status, the generation of ROS, the levels of DNA damage in the sperm nucleus and various attributes of the conventional semen profile (192, 193). Such results suggest that male infertility may have a strong psychogenic component which can be ameliorated by yoga-based lifestyle interventions aimed at reducing cognitive, as well as oxidative, stress.

### Oxidative Stress and Embryo Development

While we have traditionally viewed male reproductive competence in terms of fertilization capacity, it is now clear that the definition of “competence” should extend beyond conception to encompass the establishment of a normal viable pregnancy as well as the health and well-being of the offspring. It is therefore important to acknowledge that oxidative damage to human spermatozoa does not just influence their capacity for fertilization but also has a major impact on the developmental potential of the embryo (194, 195). Importantly, when spermatozoa are subjected to increasing levels of oxidative stress, the induction of significant DNA damage precedes the loss of fertilizing potential (196). As a consequence, it is perfectly possible for a DNA damaged spermatozoon to achieve fertilization of the oocyte. When this happens, the oocyte immediately launches into a round of DNA repair in order to address any damage in the paternal genome prior to the initiation of S-phase of the first mitotic cell division. If the oocyte makes a mistake at this point or is overwhelmed by the levels of DNA damage brought in by the fertilizing spermatozoon, it has the potential to increase mutations in the offspring that may significantly impact the latter's developmental potential and long-term health trajectory.

Oocytes are particularly vulnerable to the introduction of oxidative DNA damage by the fertilizing spermatozoon because they are deficient in the first enzyme of the base excision repair pathway, OGG-1 (197). Typically, defective human spermatozoa carry extremely high levels of oxidative DNA damage (198, 199) so the chances are, that fertilization will force the oocyte to the very limits of its repair capacity. Persistence of paternally-derived oxidized DNA base adducts into S-phase of the first mitotic division will enhance the risk of *de novo* mutations being created as the embryos enter the cleavage stage of development. Experimentally, if gametes are generated expressing high levels of oxidative DNA damage by genetically inactivating the major base excision DNA repair pathways, then the offspring exhibit high levels of *de novo* mutations, particularly G to T transversions, and live lives shortened by birth defects and disease, including cancer (200). Oxidative stress in the male germ line can therefore not only create *de novo* mutations directly; it can also generate pre-mutational damage that becomes fixed as a genetic mutation following fertilization, as a result of deficient or aberrant repair in the oocyte prior to the first cleavage division (82). It has recently been recognized that while most age-related *de novo* mutations are paternal in origin, there is also a powerful maternal contribution (83). We hypothesize that this maternal factor could be the negative impact of age on the DNA repair capacity in the oocyte (201). Viewed in this light, the creation of *de novo* mutations could be regarded, at least in part, as a collusion between the male and female germ lines. In the male germ line, we see extensive evidence of oxidative DNA damage, the incidence of damage increasing with age as the DNA repair capacity of the germ line declines. This oxidatively damaged DNA is then brought into the oocyte by the fertilizing sperm, overwhelming the latter's capacity for effective DNA repair and stimulating the creation of *de novo* mutations that ultimately impact the health and well-being, and potentially the fertility, of the offspring.

### A Unifying Concept: Oxidative DNA Damage, Infertility and Offspring Health

So, we have a male infertility landscape which, until recently has been largely unexplained. However, in the past decade or so, it has become clear that both genetics and oxidative stress play important roles in the definition of semen quality. In the final section of this review we ask whether these two epidemiological pathways might be causally linked.

**Figure 5:**
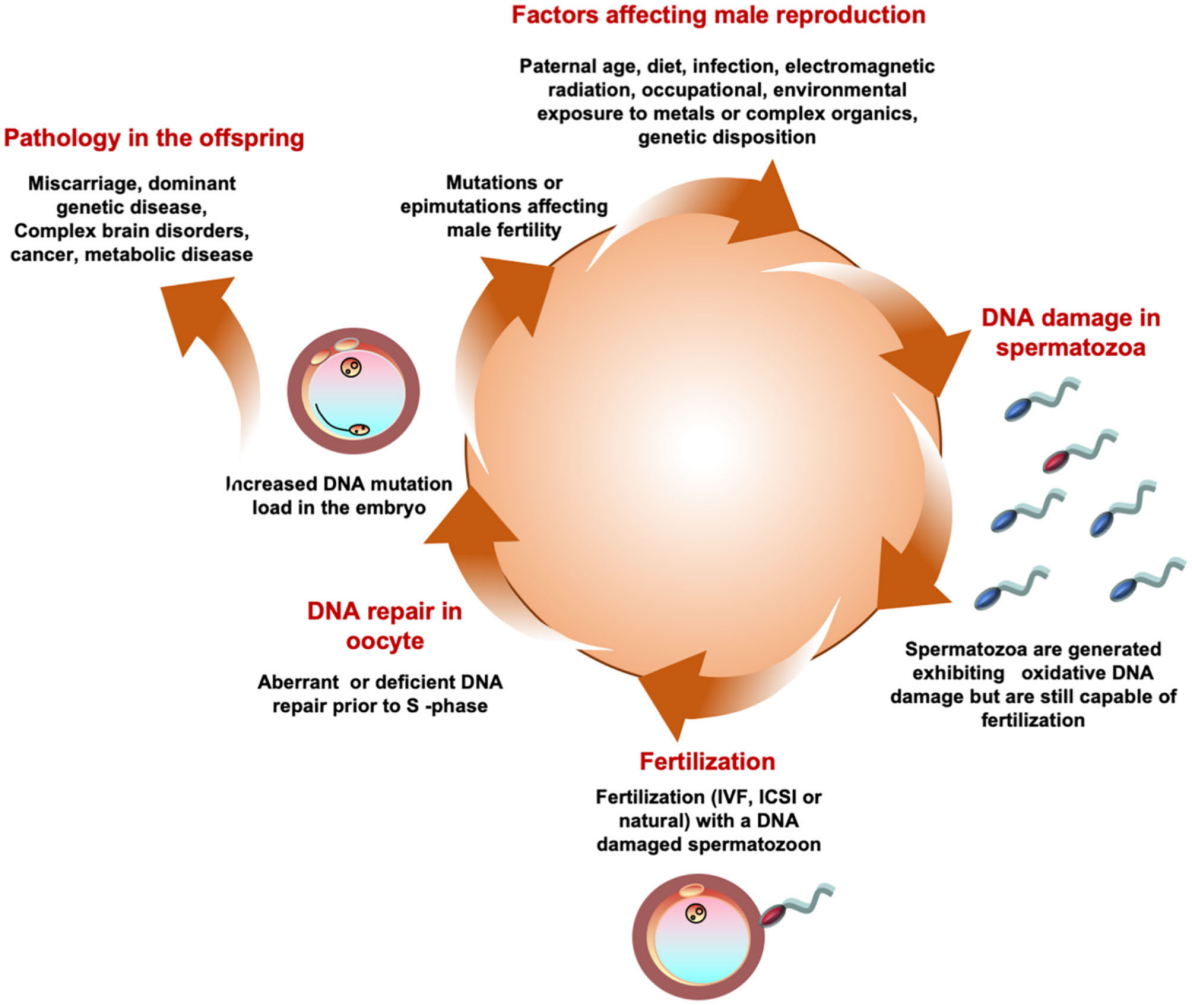
A possible unifying hypothesis. According to this model a variety of environmental, lifestyle, and medical factors conspire to generate a state of oxidative stress in the male germ. Oxidaitve stress then induces DNA damage in human spermatozoa. A spermatozoon carrying high levels of oxidative DNA damage then fertilizes the egg and in the hours between fertilization and the initiation of S-phase of the first mitotic cell division the oocyte attempts to repair the DNA damage brought in by the fertilizing spermatozoon. If the oocyte makes a mistake at this point it may fix the paternal oxidative DNA damage as a mutation in the embryo. Such mutations may induce infertility in the offspring as a result of, for example, point mutations in genes that are critical for the reproductive process or Y-chromosome deletions. Alternatively, the oxidative DNA damage may occur in an area of the genome, such as chromosome 15, that does not frequently affect fertility but influences the health and well-being of the offspring inducing pathologies, including brain disorders, imprinting errors and cancers, that are known to have a powerful paternal component.

Clearly oxidative stress in the male germ line can be driven by a great many environmental, lifestyle and clinical factors ranging from smoking, obesity, electromagnetic radiation including heat, a variety of xenobiotic toxicants including the omnipresent phthalate esters and bisphenol A, varicocele, infection and autoimmunity [(202, 203); Figure 5]. As germ cells differentiate, they become progressively less adept at DNA repair and this ineptness only increases with paternal age (204). The oxidative damage sustained by these cells will, if it is sufficiently intense, suppress the fertilizing capacity of the spermatozoa by impairing their motility and their capacity for interaction with the oocyte (85). If the damage is less intense, these cells may still retain their capacity for fertilization and thus have the ability to carry oxidatively damaged DNA (194) and, potentially, oxidatively damaged centrioles (205) and telomeres (206) (guanine rich structures at the end of chromosomes that are known to be paternally inherited and vulnerable to oxidative stress), into the oocyte, all of which could disrupt embryonic development, implantation and the progress of pregnancy to term. Genetic and epigenetic mutations induced via this mechanism have the potential to impact the fertility, health and well-being of the offspring.

In terms of infertility, the DNA fragmentation precipitated by the induction of oxidative stress has the potential to create Y chromosome deletions through aberrant recombination of the repetitive sequences that abound on this chromosome. Stochastic point mutations introduced by such means also have the potential to induce infertility if they interfere with expression of key genes involved in orchestrating the production and differentiation of spermatozoa. The complex nature of these cells ensures that there are plenty of genes to choose from.

We have recently reported that when spermatozoa are exposed to an oxidative stress, DNA damage is generated across the genome as one might have anticipated, although the sex chromosomes are to some degree protected. Against this background there is, however, one area of the human sperm genome that is particularly vulnerable to oxidative attack on chromosome 15 (207). Such susceptible genomic sites experienced a dramatic (2–15 fold) increase in their burden of oxidative DNA damage in patients undergoing infertility evaluation compared to normal healthy donors. Translocations affecting this area of the genome are known to cause male infertility (208) as are point mutations affecting genes on chromosome 15 such as SNRPA1. The latter is involved in establishing the spliceosome, a dynamic ribonucleoprotein complex responsible for orchestrating the processing of RNA during spermatogenesis, defects in which cause non-obstructive azoospermia (209). Catsper is also located in this area of the genome and defects in this critical sperm component are also associated with male infertility (210). The presence of small supernumerary marker chromosomes (sSMCs) is similarly associated with severe male infertility involving oligoasthenoteratozoospermia and most commonly involves chromosome 15 (211). Large deletions on chromosome 15 including the Catsper gene, and another gene also associated with spermatozoa, STRC (stereocilin), have also been linked to a rare condition characterized by male infertility and deafness (212). Interestingly, none of the other point mutations associated with male infertility that were covered in the first part of this review, involve chromosome 15. Whether the observed mutations are involved in the central endocrine drive to spermatogenesis, the process of spermatogenesis itself or the production of morphologically normal motile spermatozoa capable of fertilization, they occur in virtually every part of the genome but not chromosome 15. Perhaps this is too vulnerable an area of the genome to sequester genes that are critical to reproductive success.

However, chromosome 15 does encode a number of genes involved neurological development. The very area of the genome that we have found to be vulnerable to oxidative attack is also the site of genetic perturbations associated with a variety of brain disorders including Marfan syndrome, epilepsy, spontaneous schizophrenia, bipolar disease, attention deficit hyperactivity disorder and, critically, autism (213). Interestingly, all of these conditions are highly correlated with the age of the father at the moment of conception. Since paternal age is associated with oxidative stress in the male germ line, we hypothesize that an age-related oxidative attack on the paternal germ line precipitated the mutations that ultimately led to these conditions appearing in the offspring (Figure 5).

Of course, aging is just one way of creating oxidative stress in germ cells, there are many others not least of which is infertility itself. Since male infertility is commonly associated with the kind of oxidative stress we associate with aging, we might anticipate an increase in conditions such as autism in the offspring of ART patients, particularly when ICSI is used as the insemination procedure. In keeping with this proposition, a report from the USA did indeed find an increased risk of autism in the progeny when ICSI, not IVF, was used to inseminate the oocytes (214). Similarly, the area of chromosome 15 which is vulnerable to oxidative attack also houses the imprinted genes responsible for Prader-Willi and Angelman syndromes and evidence exists to suggest that both of these conditions may elevated in the offspring of subfertile couples and may be exacerbated by assisted conception therapy (215). Although this association between assisted conception therapy and imprinting disorders is not consistently observed across all datasets, the 3.44-fold increase in Prader Willi syndrome observed by Hattori et al. (215) is particularly striking and could be explained by oxidative destruction of the paternal allele, allowing the maternal allele to dominate. Interestingly, deletions in this area of the genome are thought to involve DNA fragmentation followed by aberrant recombination of flanking repeat elements (END-repeats), in much the same way as deletions are induced on the Y-chromosome (216).

Smoking is another condition associated with oxidative stress in the male germ line, that is independent of age and generates 8OHdG lesions in human spermatozoa via mechanisms that can be exacerbated by antioxidant deficient diets and OGG1 Ser326Cys polymorphisms (217, 218). There are also data to indicate a significant association between paternal smoking and cancer in the offspring, particularly leukemias including acute lymphoblastic leukemia (219). Consistent with the data presented above, it is of interest that one of the loci associated with childhood leukemia is on chromosome 15 (15q13–15) (220). Fundamentally, oxidative DNA damage induced in human spermatozoa as a result of heavy smoking is probably responsible of introducing millions of *de novo* mutations into our species (221), with implications for the future incidence of childhood leukemias (and possibly other conditions) in affected lineages.

The genomic domain we have identified as being particularly susceptible to oxidative damage is known to be a hot spot for copy number variation (222) and microdeletions in 15q11.2 BP1-BP2 (the Burnside-Butler susceptibility locus) are known to associated with intellectual impairment (223). Thus, oxidative damage to this area of the genome in human spermatozoa may be associated with a range of phenotypes in the offspring including male infertility, imprinting disorders and a plethora of behavioral/intellectual defects. Any conditions that promote oxidative damage in human spermatozoa may increase the incidence of such conditions in the offspring depending on the severity and location of the damage and the efficiency of DNA repair.

## Clinical Significance

What is the clinical significance of all this information? Fundamentally, this review is intended to highlight the potential significance of oxidative stress in both the etiology of male infertility and the mutational load subsequently carried by the offspring. The management of male infertility patients should therefore involve an assessment by the levels of oxidative stress and DNA damage suffered by the spermatozoa. If these parameters are elevated, then, and only then, should interventions be considered. The most obvious therapeutic intervention would be to give affected patients antioxidants and then monitor the levels of oxidative stress over time to ensure that this approach (or the particular antioxidant product selected) is being effective. It is important not to give antioxidants in the absence of a diagnosis of oxidative stress in order to avoid the induction of reductive stress, which can be just as damaging as its oxidative counterpart. From a preventative standpoint, it would also be important to carefully review the patient's medical history, occupation and lifestyle in an attempt to identify potential causes of the oxidative stress which could be addressed. Thus, the presence of a varicocele, infection, sedentary lifestyle, obesity, lack of exercise, poor diet, high scrotal temperatures, excessive consumption of recreational drugs including alcohol and tobacco and psychological stress, are all possible contributors to oxidative stress in infertility patients that might be addressed by simple lifestyle interventions. As far as genetic factors are concerned, the management options are more challenging because the damage has already been done in the germlines of the patients' forebearers and, in the absence of gene therapy, the only realistic option is IVF/ICSI. While this form treatment has enjoyed some success, knowing the genetic basis of the infertility (particularly for the most common genetic conditions such as cystic fibrosis gene mutations, Klinefelter syndrome, Y chromosome microdeletions, Noonan syndrome, and chromosomal translocations) may be important in counseling the patients on the likely health trajectory of their offspring and the possible benefits of using donor spermatozoa. See Ferlin and Foresta (224) for further detailed discussion of these issues from a clinical perspective.

## Conclusion

In conclusion. a variety of environmental and lifestyle factors including age, smoking, infertility, obesity, exposure to a range of xenobiotic toxicants, radiofrequency electromagnetic radiation, heat and cryopreservation (213, 225, 226) conspire to generate oxidative DNA damage and fragmentation in the male germline. Inefficient or aberrant repair then fixes this damage as a mutation, either in the germline itself or in the newly fertilized oocyte. Mutations generated in this way can cause infertility in male offspring as a consequence of Y-chromosome deletions or a wide range of mutations in other parts of the genome that influence the production or functionality of the spermatozoa. In addition, these mutations can cause a range of other diseases particularly cancer and brain disorders, including autism and spontaneous schizophrenia. This tentative unifying hypothesis is summarized in Figure 5 and provides us with a model we can test in future studies.

## Author Contributions

RJA prepared the first draft of the manuscript which was then critically reviewed by MB. Both authors approve the final version and submission of this article.

## Conflict of Interest

The authors declare that the research was conducted in the absence of any commercial or financial relationships that could be construed as a potential conflict of interest.
